# Estimating wearable motion sensor performance from personal biomechanical models and sensor data synthesis

**DOI:** 10.1038/s41598-020-68225-6

**Published:** 2020-07-10

**Authors:** Adrian Derungs, Oliver Amft

**Affiliations:** Chair of Digital Health, Friedrich-Alexander-Universität Erlangen-Nürnberg (FAU), Erlangen, 91052 Germany

**Keywords:** Biomedical engineering, Stroke, Rehabilitation

## Abstract

We present a fundamentally new approach to design and assess wearable motion systems based on biomechanical simulation and sensor data synthesis. We devise a methodology of personal biomechanical models and virtually attach sensor models to body parts, including sensor positions frequently considered for wearable devices. The simulation enables us to synthesise motion sensor data, which is subsequently considered as input for gait marker estimation algorithms. We evaluated our methodology in two case studies, including running athletes and hemiparetic patients. Our analysis shows that running speed affects gait marker estimation performance. Estimation error of stride duration varies between athletes across 834 simulated sensor positions and can soar up to 54%, i.e. 404 ms. In walking patients after stroke, we show that gait marker performance differs between affected and less-affected body sides and optimal sensor positions change over a period of movement therapy intervention. For both case studies, we observe that optimal gait marker estimation performance benefits from personally selected sensor positions and robust algorithms. Our methodology enables wearable designers and algorithm developers to rapidly analyse the design options and create personalised systems where needed, e.g. for patients with movement disorders.

## Introduction

Wearable motion sensors are frequently used in sports, medicine, and rehabilitation, to estimate gait markers for objective movement evaluation. In particular, stride count, stride duration, and stride cadence, are considered to quantify gait performance and to analyse variability across gait speed^1,2^. Moreover, gait marker analysis is employed to investigate walking disturbances in patients after stroke^3^, to evaluate sensorimotor impairments reflected in walking performance^4^, and to monitor regain of walking automaticity^5^. To date, wearable motion systems are often placed on the body, guided by intuition and practicality. Quantitative evidence of the movement characteristics across body positions is not considered. Moreover, individual performance differs according to the type of motion, e.g. running athlete vs. walking rehabilitation patient, as well as inter-individual characteristics and coping strategies, leading to unique, personal movement patterns. When left unconsidered, the variations influence sensor data. In turn, wearable motion sensors may be inaccurate in quantifying performance or health status, which results in insufficient analysis and diagnostic value. For example, Altini et al.^6^ showed that the accuracy of energy expenditure estimation during different activities, including running, is affected by the sensor placement, i.e. estimates vary depending on whether an accelerometer is worn on the chest or the wrist. Over the last two decades the benefit of wearable motion sensors in healthcare has been frequently investigated, however their integration into clinical practice is still not yet established. To reveal the full potential of wearable sensors, new methods are required that can address the key uncertainties of deployment, including the choice of wearable device position and the choice of algorithms for sensor data processing.

It has been shown that human motion varies among athletes in highly standardised walking sports and that the measurement position on the body could influence signal quality and data features, which in turn renders accurate performance estimation challenging^7^. Different scientific attempts were made to optimise motion measurements and minimise sensor positioning effects. The approaches primarily addressed the recognition of activities under varying sensor measurements, due to inter-individual differences or sensor displacement^8–13^. Kunze et al.^14^ showed that rotation-independent, frequency-domain features could mitigate wearable sensor positioning effects, therefore increase robustness in activity recognition tasks. Förster et al.^15^ proposed orientation-robust features in addition to self-calibration algorithms to overcome negative effects of sensor displacements in activity and gesture recognition. So far, investigations that studied motion variance often used measurements from physical sensors only, which restricts replication and understanding the variation effects. The practical implementation of wearable motion analysis is still done in iterative physical prototyping attempts to optimise and correct sensor positioning depending on the expected motion, which is laborious due to repeated measurements and error-prone due to variance in motion execution. Iterative analyses not only imply a burden to study participants or patients, but also require repeating the acquisition of reference information, e.g. assessment scores, with coaches or clinical experts. In turn, machine learning methods to estimate scores are evaluated in each iteration with a particular configuration of sensors only. For example, functional movement analysis in patients after stroke often builds on classification and regression methods to estimate clinician-provided assessment scores^16–18^. Repeated development and evaluation cycles delay insight and system deployment, as well as it prevents scaling up investigations, e.g. to analyse larger study populations.

A key challenge in designing wearable systems stems from the vast design space and parametrisation options, including the choice of sensor device, modalities, body positions and attachment, signal processing, and marker extraction algorithms. To find suitable wearable motion system configurations, the design space requires a systematic, model-driven analysis. Accurate biomechanical simulations have been demonstrated for human motion analysis and could provide a basis to explore and evaluate wearable system configurations. For example, OpenSim^19^ has been validated in various human and animal motion studies^20–23^. In particular, Hamner et al.^24^ investigated athlete motion and the influence of different running speeds on centre of mass acceleration. Knarr et al. analysed walking motion of hemiparetic patients to investigate effects of therapeutic rehabilitation interventions^25^. In previous work, we demonstrated that biomechanical simulation and synthesised motion data can estimate motion-related clinical assessment scores^26^, which were comparable to the analysis based on physical, wearable sensors^27^.

In this work, we build on biomechanical human models and accurate motion simulation to synthesise wearable acceleration sensor data and estimate gait markers. The methodology presented in this work can be used to rapidly gain insight in human motion variance across individuals, considering different wearable system applications. With the systematic analysis of sensor positioning effects on marker estimation performance, we can derive system design recommendations without the need for multiple, laborious measurements with real sensors. We quantify and visualise human motion performance derived from the simulated sensors to highlight suitable measurement positions. Our approach can guide wearable system design to match applications or individual characteristics, while considering hundreds of potential measurement positions across the body, including those frequently used ones. Beyond the body position, we furthermore show how our approach can support the analysis of different signal processing and marker estimation algorithms.

We present two case studies to investigate the design space for wearable motion sensor systems: Case Study 1 includes ten healthy athletes running at different speeds, Case Study 2 includes eight hemiparetic stroke survivors, who walk at self-selected speed (for details see “Methods” section). In both case studies we leverage the biomechanical motion reference from high-resolution video motion capture. Our results show that personalised or application-adapted wearable systems considering sensor position, processing parameters, and algorithm choice can significantly outperform common assumptions and design choices frequently applied in current wearable systems.

## Results

**Figure 1:**
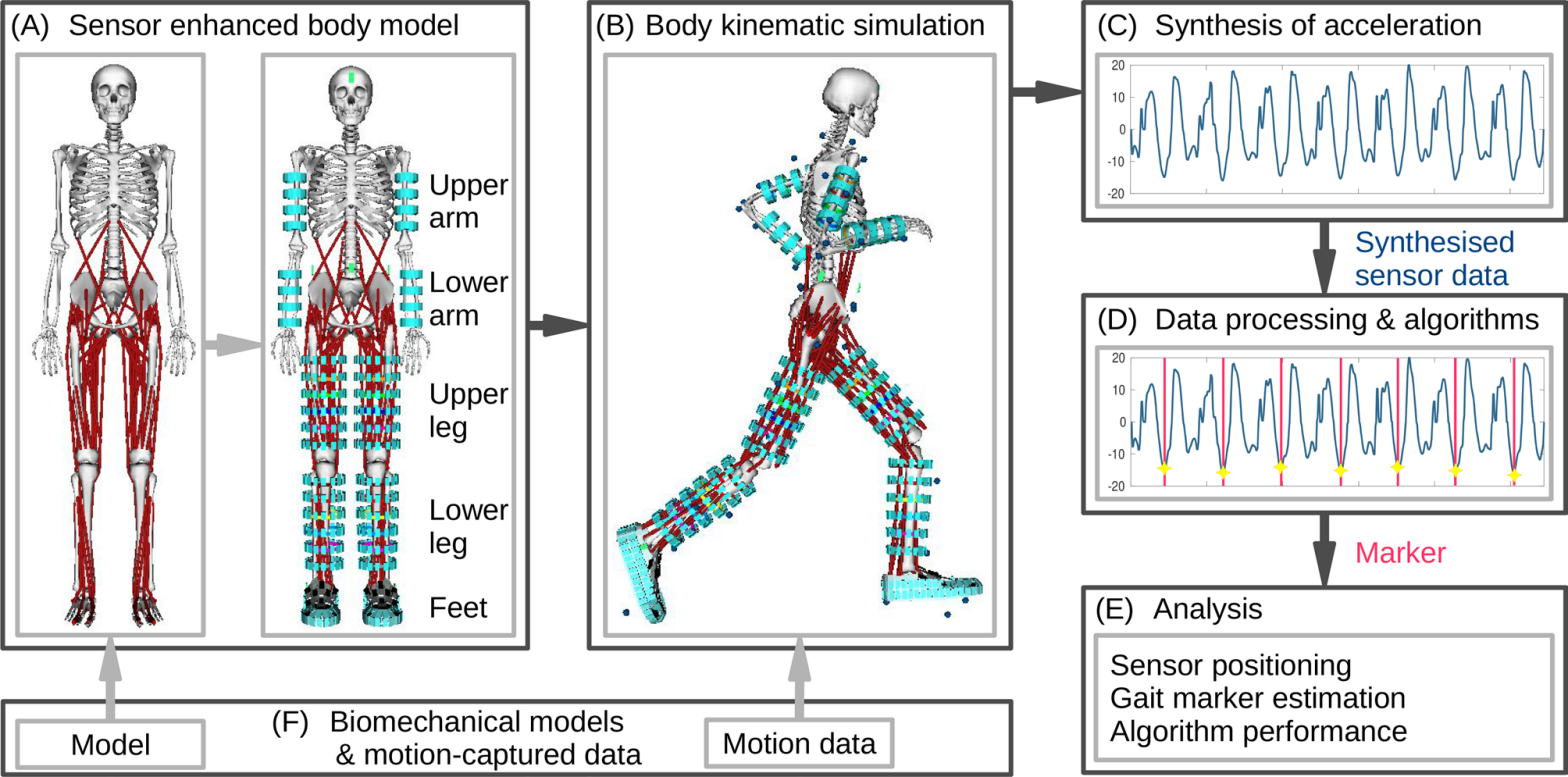
Human motion analysis using personalised biomechanical models, simulation, and data synthesis to estimate wearable system and marker performance. (**A**) Illustration of the personalised full body model, extended with motion sensor models at upper and lower body extremities. (**B**) Body kinematic simulation of the sensor-extended full body model with video motion-capture data. (**C**) Sensor data synthesis (here: acceleration) using a direct-link sensor-body attachment model. (**D**) Processing of synthesised acceleration data using gait marker estimation algorithms. (**E**) Analysis of individual movement effects on gait markers, sensor position on the body, and estimation algorithms. (**F**) Biomechanical models and video-motion data obtained from the public SimTK repository.

Figure 1 shows our methodology for personalising biomechanical models, movement simulation, and sensor data synthesis. In two case studies, including running athletes and walking patients after stroke, we illustrate marker performance in relation to wearable sensor position based on the validated biomechanical models. We demonstrate how gait markers are affected by individual human motion patterns depending on measurement position and investigate positions that provide minimal error during running and walking. In addition, we compare two gait marker extraction algorithms that use synthetic acceleration data as input and we test the sensor attachment model.

### Case study 1: Athlete running

**Figure 2:**
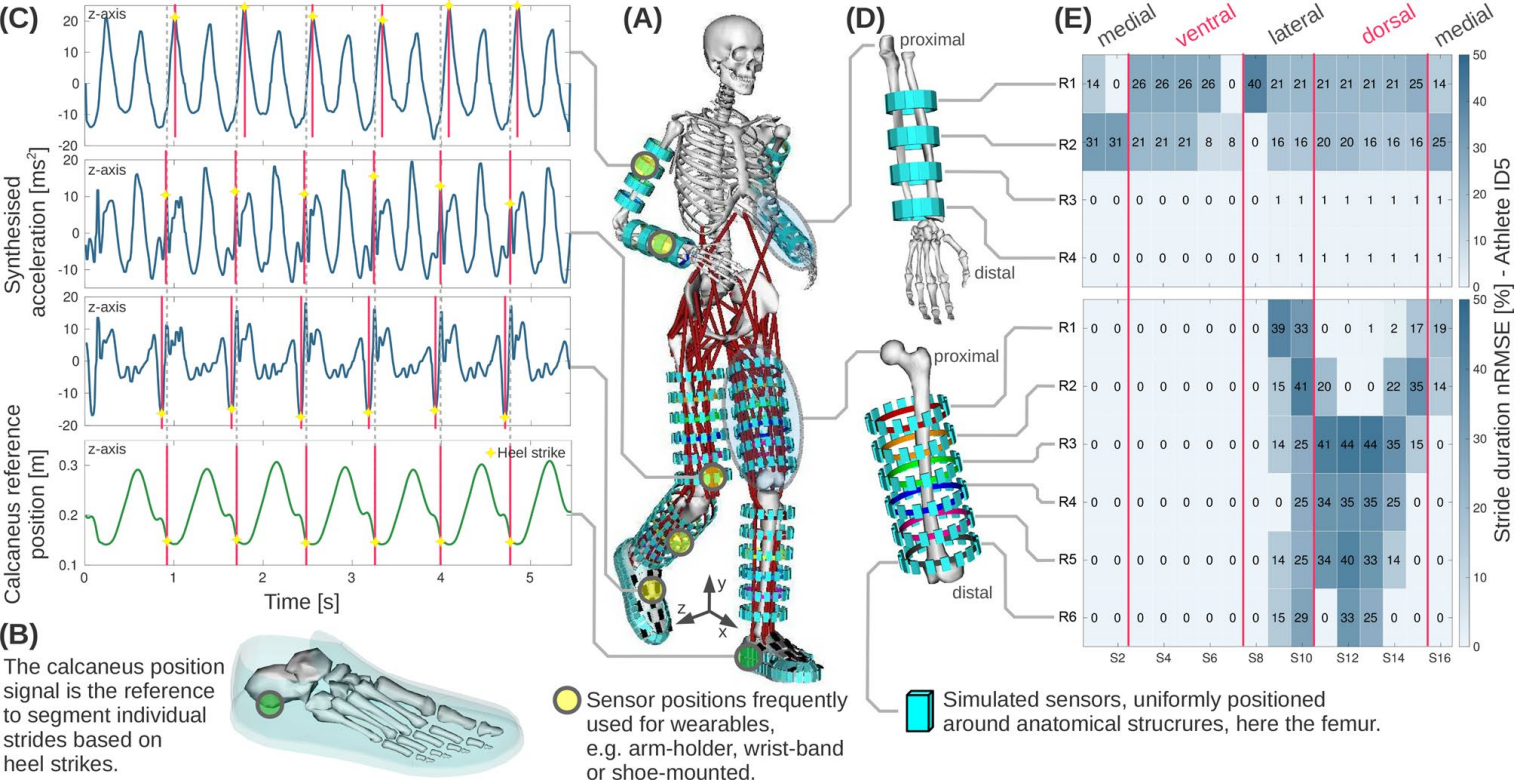
Illustration of the wearable motion analysis based on biomechanical simulations and sensor data synthesis. (**A**) Illustration of the sensor-extended, personal full-body model while running, including 834 simulated sensors uniformly positioned at upper and lower arms and legs, as well as feet. (**B**) Feet models with an inertia-free shoe model used to place sensor models. The vertical calcaneus position in y-axis orientation served as stride reference. (**C**) Example of the acceleration time series data synthesised for each simulated sensor. (**D**) Zoomed view of the lower arm (64 sensors), and the upper leg (96 sensors). For visualisation of the sensor positions, the femur was rotated. (**E**) Normalised root-mean-square-error (nRMSE) of the marker stride duration, derived between simulated sensors and the calcaneus reference. Colour-coded nRMSE maps shown here for athlete ID5.

We analysed motion data derived from 834 simulated sensors virtually placed on both upper and lower body extremities of the models of ten running athletes. We used the dataset from Hamner et al.^24^, including motion data recordings of each athlete running on a treadmill with 2 m/s, 3 m/s, 4 m/s, and 5 m/s. Figure 2 illustrates signals and performance estimates for the gait marker stride duration, depending on sensor position at the lower arm and the upper leg of one athlete. To visualise the varying marker performance, we show normalised root-mean-square-error (nRMSE) maps across body positions. Subsequently, we detail the marker estimation performance across athletes, sensor position variation, and algorithm differences.

#### Marker performance across athlete population

Stride duration estimated from simulated sensors at lower legs and lower arms showed significantly larger errors across the athlete study population and for all running speeds, compared to upper arms, upper legs, and feet on both body sides.

**Figure 3:**
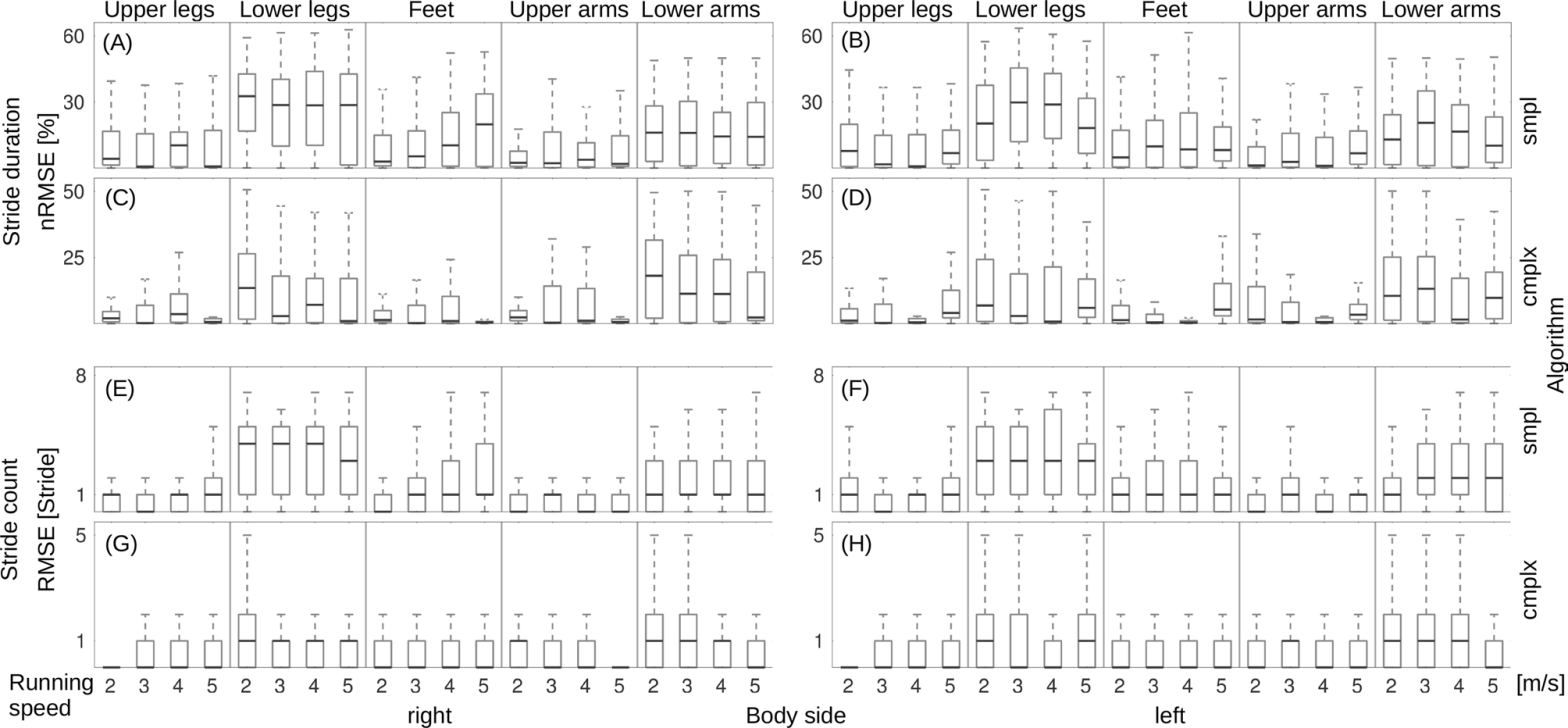
Boxplots of the error analysis for markers stride duration and stride count at various body positions averaged across all athletes: (**A**,**B**) nRMSE for stride duration, *smpl* algorithm, right and left body sides. (**C**,**D**) nRMSE for stride duration, *cmplx* algorithm, right and left body sides. (**E**,**F**) RMSE for stride count, *smpl* algorithm, right and left body sides. (**G,H**) RMSE for stride count, *cmplx* algorithm, right and left body sides.

Figure 3 shows the estimation error of the marker stride duration across all athletes, running speeds, and all simulated sensor positions. In addition, the comparison between *smpl* and *cmplx* algorithms are shown. Median error across the study population and the *smpl* algorithm was 11.0% at the right and 10.0% at left body side. For the *cmplx* algorithm, median errors across the study population were 1.3% at the right and 2.0% at the left body side. Overall, the *cmplx* algorithm yielded significantly lower nRMSE values compared to the *smpl* algorithm across all body regions and all speeds. Figure 3 shows the root-mean-square-error (RMSE) of the marker stride count. Median error estimates derived with the *smpl* algorithm were one stride for both body sides. For the *cmplx* algorithm median errors were zero strides for both body sides.

Beside stride duration and stride count, we calculated the cadence, which was expected to correlate with other markers. Non-parametric, two-tailed Spearmann test confirmed high correlations (according to Mukaka et al.^28^) between stride duration and stride cadence, $\rho = 0.82$, as well as between stride duration and stride count, $\rho = 0.72$.

#### Effects of sensor position in the athlete study

**Figure 4:**
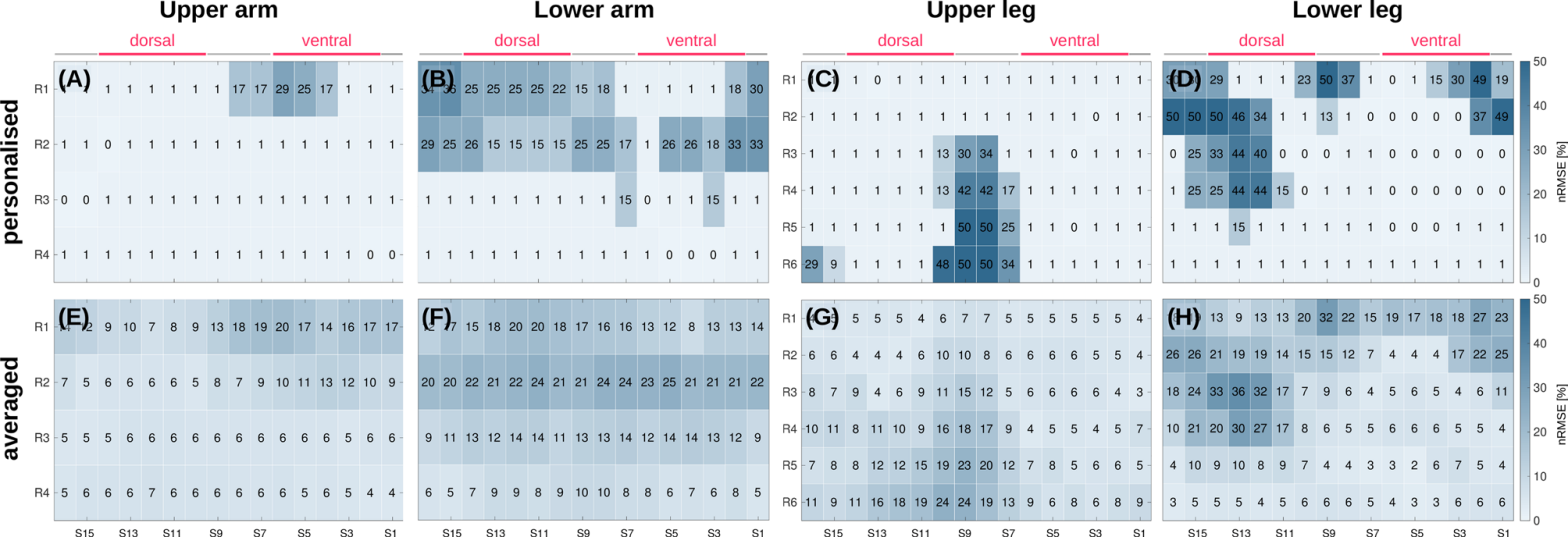
Comparison of athlete personalised (here ID10, right body side) and population averaged (ID1, ..., ID10) marker errors for stride duration, while running at 4 m/s. (**A,E**) nRMSE maps for upper arm. (**B**,**F**) nRMSE maps for lower arm. (**C**,**G**) nRMSE maps for upper leg. (**D**,**H**) nRMSE maps for lower leg.

Figure 4 shows nRMSE maps of the marker stride duration of an individual runner and the study population average for 4 m/s. More distinct error regions are visible for the individual athlete than the population average. Moreover, error maps of individual athlete and population average show different error hotspots, indicating that inter-individual differences affect the marker estimation. The error maps suggest that sensors at the upper and lower arm should be placed distal (i.e., R3–R4). At the upper leg, the average best positions are medial and ventral (i.e., S1–S6), whereas on the lower leg distal sensor positions should be considered (i.e., R5–R6).

**Figure 5:**
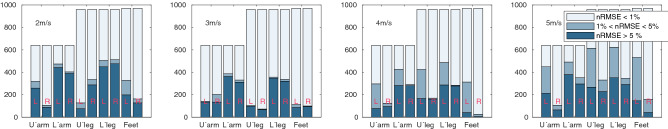
Overview on sensor position dependent stride duration estimation errors in the athlete study, including all simulated sensors at left (L) and right (R) body sides and all running speeds. The total study statistics include 640 simulated sensors at upper and lower arms, 960 sensors at upper and lower legs, and 970 sensors at each foot.

Figure 5 summarises stride duration estimation errors of all running speeds across the athlete population, including all simulated sensor positions. Errors were grouped in three levels: below 1%, between 1 and 5%, and above 5%. For running speeds between 2 m/s to 4 m/s and sensor positions on upper arms, upper legs, and feet, errors were below 1%. In contrast, sensor positions at lower arms and lower legs showed larger error, exceeding 5%. At a running speed of 5 m/s, the total number of sensor positions with errors above 5% increased, even for the upper legs. While errors at feet increased with speed too, errors above 5% remained almost unchanged. SI Figure S10 (see supplementary material) illustrates the error distribution per athlete, considering all simulated sensors and all running speeds.

Table 1 summarises stride durations derived from the best simulated sensor using the cmplx algorithm at each body region and the calcaneus reference. We derived the heel strike reference from the vertical position of the calcaneus during the ground contact and segmented individual strides accordingly (see Fig. 2). Stride duration std. dev. at the calcaneus reference is approx. 100 ms. Relative errors between calcaneus reference and simulated sensors increase with running speed. For the athletes investigated in this study, left and right body side show similar nRMSE, indicating that gait patterns were symmetric. The analysis furthermore shows that for the best individually selected sensor position across all athletes nRMSE will not exceed 11.6%, i.e. 86 ms for any body region and running speed. In contrast, the worst sensor positions yield nRMSE up to 54%, i.e. 404 ms at the lower leg at 5 m/s. In particular, for the selected athlete (ID10), the best sensor results in zero nRMSE, where as the worst sensor results in 51% nRMSE, or approx. 400 ms.

To further illustrate the effect of sensor position variation on estimated stride duration, we derived stride distance errors of the best sensors. Average distance errors at 5 m/s were 4.88 ± 1.53 cm for the upper arm, and below 5.52 ± 1.53 cm at the lower arm, upper leg, lower leg, and foot at both body sides. Below 5 m/s, errors decrease to below 3.36 ± 0.27 cm at 4 m/s, below 2.81 ± 0.21 cm at 3 m/s, and below 1.64 ± 0.13 cm at 2 m/s at both body sides.

**Table 1:** Stride duration reference and best personal sensor position per body part across all athletes derived with the cmplx algorithm.

Speed (m/s)	Calcaneus Reference		Best simulated sensor															Body side
			Upper arm			Lower arm			Upper leg			Lower leg			Foot			
	Mean	SD	Mean	SD	nRMSE	Mean	SD	nRMSE	Mean	SD	nRMSE	Mean	SD	nRMSE	Mean	SD	nRMSE	
	(m/s)		(m/s)		(%)	(m/s)		(%)	(m/s)		(%)	(m/s)		(%)	(m/s)		(%)	
2	814.6	96.4	813.8	97.2	0.1	814.7	96.5	0.0	814.5	96.5	0.0	814.6	96.4	0.0	814.7	96.5	0.0	Right
3	794.0	93.4	774.0	90.3	2.5	772.9	90.6	2.7	773.4	90.5	2.6	773.4	90.5	2.6	773.3	90.5	2.6	
4	772.3	94.4	727.6	83.6	5.8	728.7	84.2	5.6	728.9	84.4	5.6	728.9	84.6	5.6	728.8	84.5	5.6	
5	743.7	101.3	657.6	69.8	11.6	658.0	69.7	11.5	657.8	69.7	11.5	657.8	69.6	11.5	657.6	69.7	11.6	
2	813.0	98.2	814.6	97.6	0.2	808.4	98.5	0.6	813.0	98.2	0.0	812.8	98.2	0.0	813.3	97.9	0.0	Left
3	793.5	94.3	758.2	77.8	4.4	773.1	90.6	2.6	773.8	90.8	2.5	773.9	90.9	2.7	773.9	90.8	2.5	
4	770.7	96.1	728.4	86.7	5.5	726.7	84.2	5.7	727.0	85.9	5.7	725.8	86.5	5.8	727.5	85.3	5.6	
5	744.3	101.0	658.8	70.8	11.5	658.2	73.7	11.6	658.2	71.8	11.6	662.2	71.2	11.0	660.3	71.3	11.3	

Mean, std. dev. (SD), and relative error (nRMSE) between best personal simulated sensor position and the calcaneus reference are shown for each running speed. With speed, errors increase, but personal sensor positioning can limit relative error to below 11.6%.

#### Stride duration algorithm performance in athlete study

**Figure 6:**
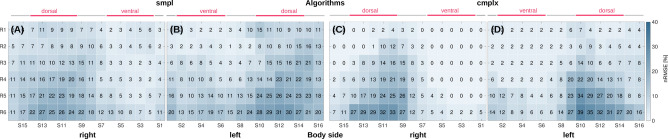
Algorithm comparison for stride duration estimation based on nRMSE maps averaged over all running athletes (running speed 5 m/s, right and left upper leg). (**A**,**B**) *smpl* algorithm. (**C**,**D**) *cmplx* algorithm.

Figure 6 shows nRMSE maps of the marker stride duration and sensor positions at the upper legs averaged across all athletes. In general, the *cmplx* algorithm yielded wider area with low nRMSE compared to the *smpl* algorithm. SI Figure S11 (see supplementary material) shows the nRMSE maps for both algorithms and a selected athlete.

### Case study 2: Walking patients after stroke

To further explore our methodology, we analysed the gait marker stride duration of patients with a hemiparesis due to stroke. In this case study, we focus on upper legs and synthesise data from 384 simulated sensors positions, 192 simulated sensor positions per upper leg. We focus on upper legs in Case Study 2 due to the following reasons: (1) earlier clinical studies demonstrated that wearable motion sensors at the upper legs were suitable to investigate patient movement and different activities of daily living during therapy and free-living^29,30^. (2) Case Study 1 revealed that the upper legs yield significantly lower marker estimation errors compared to the lower legs (see Fig. 3). In Case Study 2, we double the spatial density of the simulated sensors compared to Case Study 1, to investigate error distribution in more detail. Our analysis is based on freely available motion data of eight hemiparetic patients after stroke, where patients walked with a self-selected speed on a treadmill before and after an intervention therapy^25^. Hence, recordings before the therapy intervention are denoted as *pre*, the recording subsequent to intervention as *post*. Based on the results of Case Study 1, we used the *cmplx* algorithm to analyse the gait marker stride duration in the patient study data.

#### Affected vs. less-affected body sides in *pre-* an *post-*intervention

**Figure 7:**
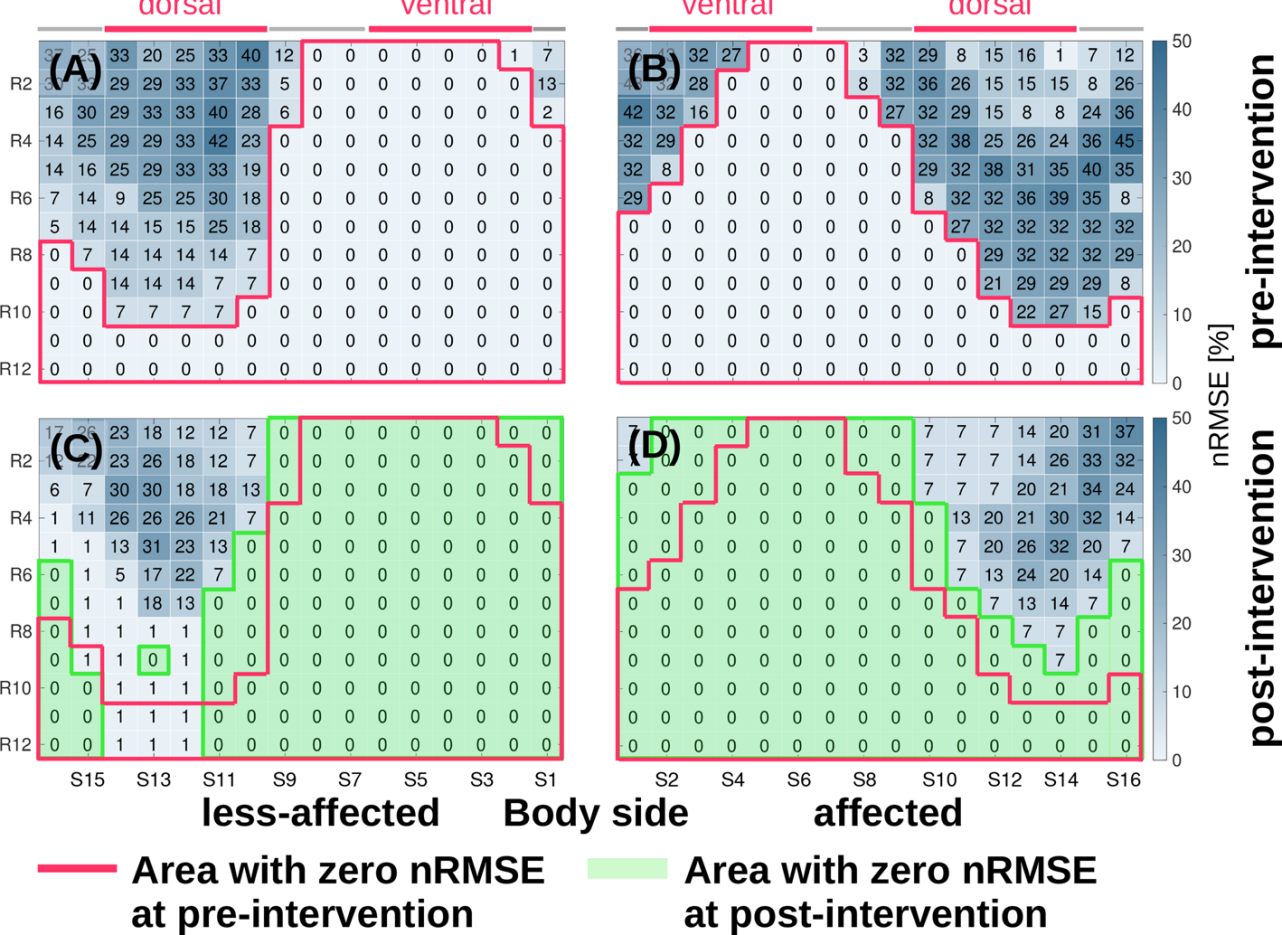
nRMSE maps for marker stride duration of a hemiparetic patient (ID8, left side affected). Walking speed was 0.3 m/s and 0.5 m/s respectively in pre- and post-intervention data. (**A**,**B**) Less-affected and affected upper leg for pre-intervention. (**C**,**D**) Less-affected and affected upper leg for post-intervention. Both body sides show larger error-free areas after the intervention. Ventral sensor positions should be favoured over dorsal ones.

Hemiparetic patients after stroke suffer motor impairments, which typically limit functional capabilities asymmetrically between body sides, leaving patients with an affected and a less-affected body side. Patients adapt to functional limitations and develop individual compensation mechanisms. Especially for those individual movement compensation, personalised wearable systems with tailored sensor positions are of interest. Figure 7 shows nRMSE maps for simulated sensor positions at the affected and less-affected upper leg for pre- and post-intervention data of one patient. The less-affected upper leg yields a larger error-free area at the ventral and medial side compared to the affected side. Comparison between pre- and post-intervention show that the error-free area increases for both body sides in post-intervention.

#### Personalised sensor positions in patient study

**Figure 8:**
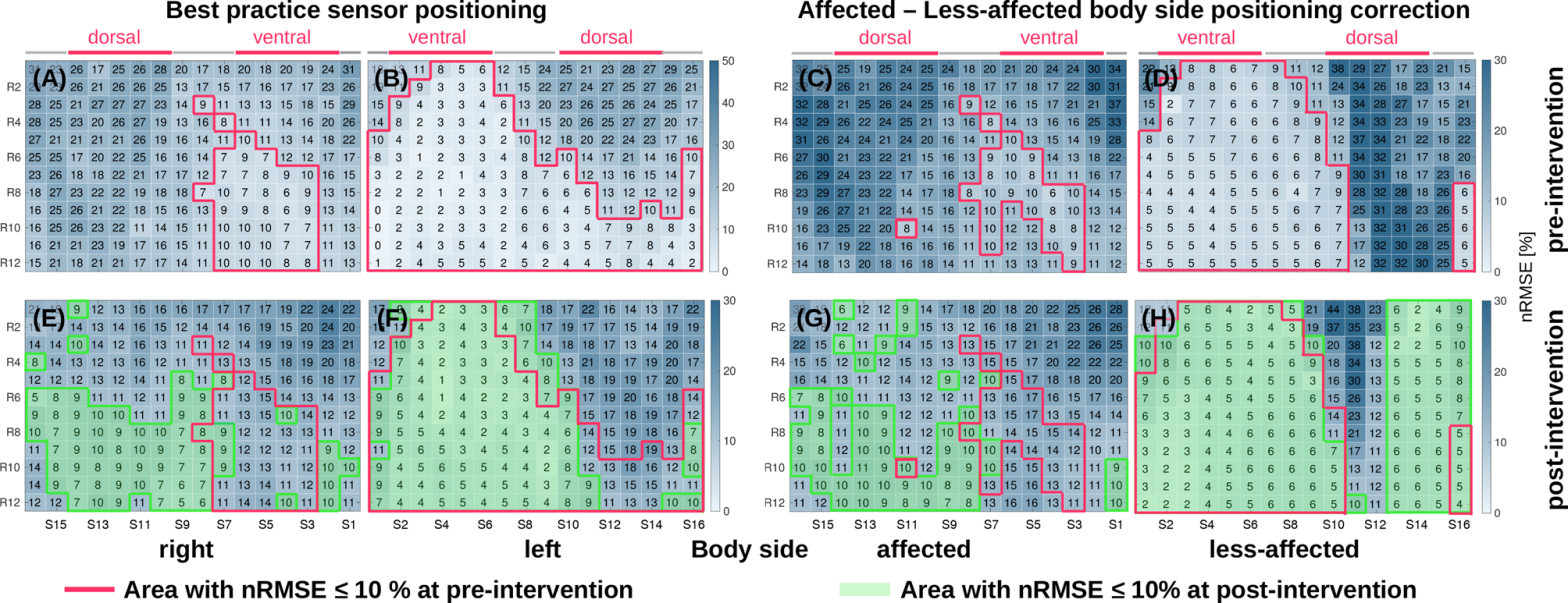
Average estimation error of stride duration depending on sensor position across all eight hemiparetic patients derived for pre- and post-intervention. (**A**,**B**,**E**,**F**) nRMSE maps for left/right upper leg region sensor placement, ignoring the impaired body side. (**C**,**D**,**G**,**H**) nRMSE maps when correcting for affected and less-affected body sides.

A common “best practice” is that sensors are symmetrically positioned on each body side, which ignores the functional differences in hemiparetic patients. Figure 8 illustrates effects of simulated sensor positions at left and right body side compared to positions corrected for each patient’s affected and less-affected sides. To better quantify the study intervention effect, we highlight nRMSE map areas with an nRMSE $\le$ 10%. Six out of eight patients, had an affected right side, which is influencing the right body nRMSE map shown as a comparably small region of nRMSE $\le$ 10%. Correspondingly, the left body nRMSE map was mainly derived from strides of the less-affected side, which reflects in the larger region of nRMSE $\le$ 10%. When correcting for affected and less-affected body sides, the less-affected side showed a ventral area with nRMSE $\le$ 10% prior to the intervention. In comparison, the affected side had a smaller region of nRMSE $\le$ 10%. After the intervention regions with nRMSE $\le$ 10% increased for both body sides while for the affected side a shift of the region to the medial-dorsal was observed.

We analysed stride duration derived from the best and worst simulated sensor position at the upper leg to investigate the nRMSE range, when correcting for affected and less-affected body sides. Results are summarised in Table 2. Across the patient population, the best simulated sensor showed nRMSE below 0.25%, where correcting for affected and less-affected body sides had negligible effect on estimation performance. In contrast, stride duration nRMSE went up to 44.26% for the worst performing simulated sensor position. For the best sensor positions, nRMSE based on pre-intervention data were higher at the affected (avg. 0.24%) than the less-affected side (0.01%). From post-intervention data, we observed similar nRMSE on the affected (avg. 0.08%) and less-affected side (avg. 0.03%).

The sensor counts with a nRMSE below 10% were 19 and 43 sensors for pre- and post-intervention at the affected side. For the less-affected side, 109 and 163 sensors had a nRMSE below 10% at pre- and post intervention. SI Figure S12 (see supplementary material) shows the sensor count statistics with nRMSE below 10%. Sensor count differences are significant for the less-affected side between pre- and post-intervention ($p=0.035$). nRMSE across the study population showed significant differences between affected and less-affected body sides as well as for each side between pre- and post-intervention.

**Table 2:** Comparison of best and worst performing simulated sensor position to estimate the gait marker stride duration in the patient study.

Body side	Calcaneus reference		Upper leg-simulated sensor						Body side corrected	Calcaneus reference		Upper leg-simulated sensor						Rehab intervention
			Best			Worst						Best			Worst			
	Mean	SD	Mean	SD	nRMSE	Mean	SD	nRMSE		Mean	SD	Mean	SD	nRMSE	Mean	SD	nRMSE	
	(ms)		(ms)		(%)	(ms)		(%)		(ms)		(ms)		(%)	(ms)		(%)	
Right	1447.4	397.7	1443.9	401.2	0.2	810.1	276.1	44.0	Less-affected	1242.9	372.2	1242.8	372.2	0.0	713.6	167.8	42.6	Pre
	1243.6	376.9	1243.3	371.9	0.0	698.1	127.6	43.9		1247.5	374.7	1247.9	374.7	0.0	718.7	189.1	42.4	Post
Left	1443.6	397.4	1444.8	397.1	0.1	809.7	198.5	43.9	Affected	1446.8	400.8	1443.3	400.8	0.2	806.4	279.3	44.3	Pre
	1246.9	374.7	1247.4	375.0	0.0	734.3	216.5	41.1		1444.2	397.6	1445.4	397.6	0.1	813.3	194.0	43.7	Post

Mean, std. dev. (SD), and relative error (nRMSE) of all strides were derived for both body sides and corrected for affected and less-affected sides in pre- and post-intervention, respectively.

### Sensor attachment

To investigate and quantify effects that the sensor attachment would induce, e.g. attachment at soft tissue, we extended the direct-link attachment and simulate conditions that would lead to particularly large effects on motion sensor data and gait markers. We investigate the upper leg, where soft tissue artefacts are considered large^31^ and apply an additive motion noise model.

On average, the comparison between synthesised acceleration with and without additional motion noise resulted in negligible differences in the stride duration of athletes and patients. In Case Study 1, max. difference in stride duration was 3 ms (0.4% of avg. stride duration) for the best and 70 ms (9.6% of avg. stride duration) for the worst performing sensor position. For patient walking data in Case Study 2, differences remain below 38 ms (5% of avg. stride duration).

## Discussion

Wearable technology is facing a variety of challenges in evaluating designs and algorithms, human-centred design, and personalisation^32,33^. We believe that our methodology can provide a major step towards rapid and systematic design exploration, including software and hardware, as well as system personalisation. We introduced a model-based methodology to systematically investigate the estimation performance variation in frequently considered gait markers, stride count, stride cadence, and stride duration, depending on sensor positioning and algorithms. For the first time, a simulation based on biomechanical and sensor models and sensor data synthesis is analysed. We consider that the model and simulation-driven approach brings considerable advantages over physical movement analysis.

In Case Study 1, our approach demonstrated that increasing running speed in athletes increases marker estimation error from 0.1 to 11.6% on average. We investigated body positions that are frequently considered by runners for wearing sensor devices, as shown in Fig. 2, including the upper arm (e.g. music player or smartphone in an arm-holder), the lower arm (e.g. wrist-worn devices, smartwatches), the foot (e.g. for shoe and shoe-tongue integrated sensors), as well as the upper and lower leg (e.g. for wearable device straps). Estimates at upper legs, feet, and upper arms showed lower average nRMSE (0.2%) compared to simulated sensors at lower legs and lower arms (18.1%).

Our analysis highlights that not the single lowest-error sensor position leads to optimal marker estimation, but rather choosing a body segment or region with overall low error. For example, wearable sensors at the wrist, e.g. smartwatches, could yield minimal marker estimation error during running when worn at the optimal position. However, error increases rapidly, even for minor position variations around the optimal one, e.g. due to sliding of the smartwatch along the arm. In direct comparison, an upper arm holster not only attains similar minimal error estimates as the wrist-worn smartwatch at optimal positions, but moreover retains lower error for position variations (see Fig. 4E,F). A similar relationship holds for upper and lower legs. Thus, our results reveal that placing and wearing sensors at upper extremities can be less cumbersome and laborious to match and maintain optimal performance than at the lower extremities. With the goal to understand body side differences, we focused here on frequently used wearable device positions at extremities, i.e. arms and legs. For athletes, analysing motion asymmetry is a long-standing scientific topic^34^. Our results show that running athletes may attach devices to either body side since the gait markers estimation performance is similar. As our analysis of 834 simulated sensors in the full-body model demonstrates, there are no constraints regarding the analysis of wearing positions. Thus, the approach can be applied to central body positions, too. Our present results warrant investigations of further temporal and spatial gait features while simultaneously acquiring reference data. In particular, reference data for other common gait parameters, including toe-off, single- and double-stand support, and ground contact time may be derived to analyse suitable algorithms and sensors.

With Case Study 2, we showed that in hemiparetic patients after stroke, nRMSE of stride duration can vary substantially between body sides. In particular when correcting for the patients’ affected and less-affected body sides, asymmetric error maps were observed. Clearly, the less-affected body side shows wider regions with low nRMSE than the affected side, thus is better suited for reliable gait marker estimation. nRMSE map changes from *pre-* to *post-*intervention may illustrate the patients’ progress over time and altered movement capabilities. Even though the rehabilitation therapy period was only 12 weeks, consistent changes in nRMSE of stride duration indicate that fixed sensor positions during recovery are insufficient to capture subtle movement changes. It is likely that changes will be caused by altered compensation mechanisms and depend on individual neurological condition and plasticity^35,36^. Therefore, changes in gait marker nRMSE maps during the recovery can signal that wearable sensor systems should be adjusted.

The nRMSE maps show that regions with nRMSE $\le$ 10% grow from pre- to post-intervention, which may indicate that walking inconsistencies decrease. Indeed, walking performance was reported to improve, as walking speed and muscle activation increased for all patients^25^. Furthermore, our analysis suggests that wearable motion sensor systems should be personalised, i.e. select sensor position that maximises performance for an individual wearer. For example, optimal sensor position at the upper leg of the affected body varies inter-individually along the ventral and dorsal side between 6 and 27%. A consequence is that symmetric sensor position across body sides—although intuitive and applied in clinical measurements—will not yield optimal, body-side specific gait marker estimations. To analyse side differences, sensor position should be adjusted to individual patient motion patterns.

In both case studies, motion data that have been recorded during running and walking on a treadmill were analysed. It is known that the biomechanical dynamics and movement pattern on a treadmill are different from overground walking and running^37^, hence sensor positioning during overground walking should be investigated for potential remote monitoring application in sports and outside the clinic.

The central contribution of this work is a novel model and simulation-driven methodology to investigate wearable sensor systems based on biomechanical and sensor models, and sensor data synthesis. The simulated sensors and sensor data are the basis to investigate relative differences in gait marker estimation and algorithm performance. The approach builds on the quality of motion data and models. For example, it is known that non-optimal marker placement could compromise biomechanical simulations due to soft tissue movement^31,38,39^. However, motion data measurements and analysis by Hamner et al. and Knarr et al., were based on accurate motion tracking (Vicon$^\circledR$ system) and an established biomechanical modelling workflow (OpenSim). Optical markers were placed on rigid body positions, i.e. joints, where soft tissue is minimal. Further, OpenSim provides an inverse kinematic modelling to derive accurate motion simulations that match video data^19^. While the models could be further validated to derive absolute marker estimates, our approach demonstrates that the relative differences provide a wealth of insight already.

For the sensor model, we assumed a direct-link sensor-body attachment, intended to maximise the fidelity at which body motion is reflected in sensor data. Hence, our analysis considered ideal sensor data and actual marker estimation errors may get larger. To account for any no-direct sensor-body attachment, e.g. considering muscle and other tissue, or clothing^40^, requires additional attachment model assumptions, which we avoided in favour of focusing on the basic marker estimation methodology. Similarly, we deployed a non-deforming shoe model to guide sensor positioning, but avoided specific design assumptions. Shoe design and mechanical parameters vary greatly, including materials, closing mechanisms, and similar, thus require further analysis. Our quantitative analysis of soft tissue attachment therefore considered a basic motion noise model at the upper leg. Our evaluation indicates that the extent at which a soft tissue attachment affects the gait marker estimation ranges approx. between less than 1% and less than 10%. While we conclude from the analysis that our present approach is viable, further development of attachment models may support quantification for various body conditions and clothing options.

Previous work has shown that the clinical scores of the Lower-Extremity Fugl–Meyer Assessment estimated with machine learning algorithms and time–frequency features are not affected by varying motion sensor orientation, i.e. rotations up to 15$^{\circ }$^26^. Hence, there is a clear potential for adequate algorithms to compensate at least part of the motion sensor positioning error. Our present analysis confirmed that the gait marker estimation error could be significantly reduced by adding signal processing, i.e. moving from the smpl to the cmplx algorithm. The method presented here will thus help in further research and development to investigate the balance of sensor position, unattended position errors that may occur over time, and computational complexity of estimation algorithms. Similarly, the design space of digital biomarkers, e.g. to describe movement or range of motion of the knee or hip, can be interpreted as a trade-off analysis between sufficient resolution to represent clinically important changes in the digital biomarker and the required wearable system complexity. Clinical practice calls for methods to track individual changes in patients^41^. We consider our findings to be an important step towards personalisation and to lower the effort for creating individualised diagnostic tools. Clearly, the fundamental methodology of our approach is not limited to motion analysis and inertial sensors only. We believe that the approach can be extended to other human physical and physiological phenomena and to model and simulate further sensor modalities, for example, to describe muscle activation by electromyography.

With the presented methodology, motion data is synthesised from motion of validated biomechanical models, which can be advantageous over motion analysis methods based on deep learning data models^42–45^. Generating spatio-temporal human motion patterns with data models requires large datasets and tailored training processes^46–49^. Obtaining accurate motion representations from data models is particularly challenging for pathological gait, i.e. gait patterns accounting for motion compensation, as it is observable in patients after stroke. Besides individual anatomical features, including muscles, joints, and ligaments, simulating forward or inverse kinematics are computationally expensive. So far, only rigid body models were approximated with data models at the expense of accurate biomechanics.

In conclusion, our approach explores how personalised biomechanical models and sensor models could complement human movement analysis and wearable system design. With the simulations and motion sensor data synthesis, our methodology enables wearable designers and algorithm developers to analyse any measurement positions at the human body and find best suited device positions, algorithms, etc. The results of this investigation open a new avenue for digital twin models in wearable system planning and performance estimation before physical prototypes are made. Both, movement-related sports and healthcare applications will benefit from the methodology to create rapid insights in wearable device performance and optimisation options for hardware and software. Our methodology provides the foundations to design wearable systems to individual needs, e.g. to track movement irregularities between body sides or athlete-specific coping strategies, hence maximising opportunities for movement performances optimisation. Moreover, stroke rehabilitation and other fields of motion-related diseases could benefit from our method, i.e. to tailor wearable systems to patients and thus transfer detailed human movement analysis to out-of-lab applications.

## Methods

### Case studies

We demonstrate in two case studies how sensor positioning influences the estimation of gait markers. Publicly available datasets are considered by Hamner et al.^24^ and Knarr et al.^25^. The methods presented in this work were carried out in accordance with relevant guidelines and regulations and all experimental protocols were approved by the FAU ethics committee. All subjects signed an informed consent. For Case Study 1^24^, data from ten athletes during running with 2 m/s, 3 m/s, 4 m/s, and 5 m/s on a treadmill were used. The runners were healthy men (age: 28 ± 5 years, height: 1.77 ± 0.04 m, body weight: 70.9 ± 7.0 kg). For Case Study 2, data from eight hemiparetic patients, including three men, during walking (age: 63 ± 8.6 years) with chronic stroke (more than 6 months post-stroke) were used. Patients received a movement training intervention and were recorded before, i.e. *pre-*, and after, i.e. *post*, intervention. Patients walked with self-selected treadmill speeds for both recordings. For both datasets, motion data and kinematics were recorded using a Vicon$^\circledR$ motion-capture system.

### Biomechanical models and simulation

We used OpenSim (OpenSim, Version 3.3, Simbios, Simbios/SimTK, CA, USA) to simulate biomechanical models during running or walking. For Case Study 1, we used a 12 segment, 29 degree-of-freedom generic musculoskeletal anthropometric scaled full-body model (*FullBody*)^22^, where lower extremity and back joints were driven by 92 Hill-type musculotendon actuators. Arm motion was driven by torque actuators, according to Hamner et al.^50^. For Case Study 2, we used a lower-body-torso-head model based on the 23 degree-of-freedom musculoskeletal human model (*Gait2354*)^51^, including 54 musculotendon actuators of the lower body, torso, and head.

### Simulated sensor positions

**Figure 9:**
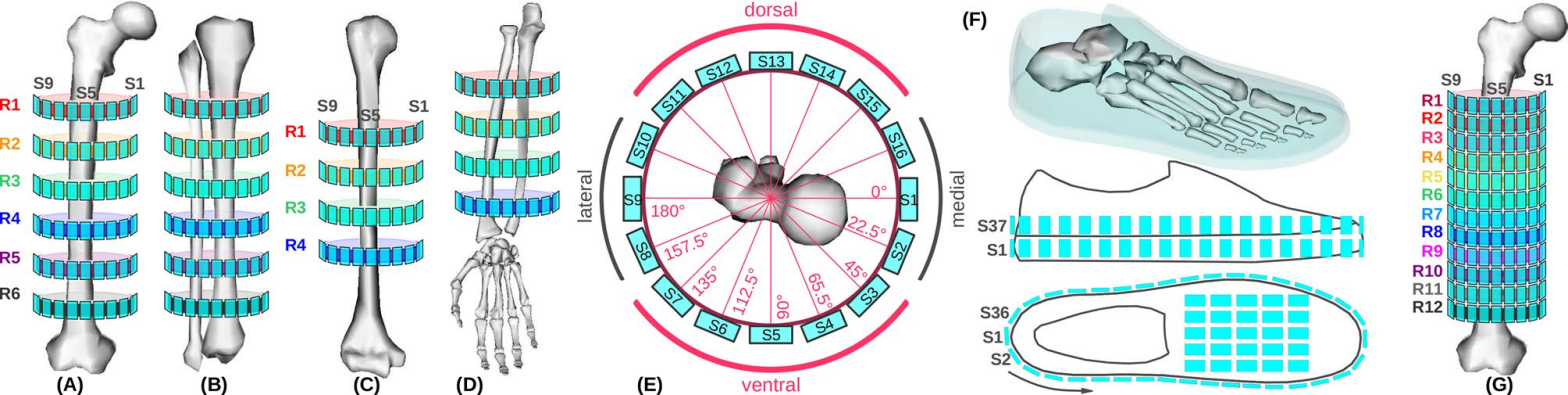
Illustration of simulated sensors. Coloured ring numbers ($\textsf {Rx}$) indicate vertical position along the bone. Designators $\textsf {Sx}$ show identification numbering around objects, where only visible sensors are indicated. (**A**) Femur in ventral orientation. (**B**) Tibia and fibula in ventral orientation. (**C**) Humerus. (**D**)Radius/ulna. (**E**) Per ring, 16 sensor positions are uniformly distributed, shown in top-view for the femur. (**F**) Sensor positions at the virtual shoe, including two rows with a total of 72 sensors and 25 additional sensors on the instep. (**G**) Femur for Case Study 2, where 12 rings with a total of 192 simulated sensors at each upper leg are considered.

The biomechanical models were extended by simulated sensor models. Figure 9 illustrates simulated sensor positions on the body. The *FullBody* model was extended by a total of 834 simulated sensors, uniformly distributed on upper and lower arms, upper and lower legs, and feet. The *Gait2354* model was extended by a total of 394 sensors. To investigate subtle movements, i.e. due to potential motion compensation mechanisms of the patients, we simulated 192 sensors on each upper leg, which is double the amount of simulated sensors used in the athletes study.

### Sensor model and data synthesis

We defined the sensor attachment model as direct-link (*WeldJoint*) between simulated sensor and bone in the specification of both biomechanical models, *FullBody* and *Gait2354* models. Both models contain parameter definitions of the musculoskeletal structure and biomechanical properties of bones, joints and muscles. Sensors were designed as 5 mm$^3$ cubes without inertia, and scaled in volume by factors of 0.001, 0.005, and 0.003 in x-, y-, and z-axis, respectively. Using parametrised coordinates with respect to anatomical structures, virtual sensors were uniformly positioned on both body sides. Using kinematic simulations of biomechanical models, we derived position trajectories of all simulated sensors in Cartesian coordinates: x-axis (anterior-posterior), y-axis (vertical), and z-axis (lateral). Subsequently, y-axis acceleration was synthesised ($\vec {\mathsf{A}}_{\mathsf{S}}$) with a sampling rate of 100 Hz, by calculating the second derivative of the position trajectory as $\vec {\mathsf{A}}_{\textsf {S}} = \frac{\textsf {d}^\textsf {2} \vec {\mathsf{p}}}{\textsf {dt}^\textsf {2}}$, where $\vec {\textsf {p}}$ refers to the simulated sensor position trajectory^26^. Sensor data synthesis were performed with MATLAB (MATLAB, Release 2017b, The MathWorks, Inc., Natick, MA, USA).

### Data processing and algorithms

Synthesised sensors were further processed to derive the gait markers using two algorithms, denoted as *smpl*, and *cmplx*. In the *smpl* algorithm, gait markers were derived without further processing from the synthesised acceleration signal of each simulated sensor. For the *cmplx* algorithm, we first filtered the synthesised sensor signal with a Savitzky–Golay filter (5th order, 25 samples window length). To segment individual strides, we applied MATLAB’s *peakfinder* method in both algorithms. Next, we calculated the stride duration, stride cadence, and stride count. Stride duration was computed as time between two detected heel strikes (peaks) from the synthesised acceleration of the simulated sensor as well as the calcaneus reference. Stride count was derived by counting the peak periods and stride cadence as strides per minute. We derived stride distance errors as a spatial difference between stride distances of the calcaneus reference and simulated sensors. To obtain the stride distance errors, we first derived the temporal difference between heel strikes of the calcaneus and each simulated sensor and subsequently mapped the time to distance according to the leg’s anterior–posterior (x-axis) motion.

### Gait marker evaluation

We derived a gait reference from the vertical position trajectory of the calcaneus for each study participant in both case studies using the unfiltered position data. Marker estimation errors were derived as nRMSE corresponding to ${\textsf {nRMSE}} \, = \, \frac{\sqrt{{\textsf {(M - C)}}^2}}{\textsf {C}} \, \times {\textsf {100}}$, where $\textsf {M}$ refers to the estimated gait marker from the simulated sensor and $\textsf {C}$ refers to gait marker according to the calcaneus reference. In Case Study 1, between four and seven strides were analysed per athlete and running speed. In Case Study 2, between eight and eleven strides per patient were available in pre- and post-intervention. All statistical analyses used MATLAB’s implementation of the Wilcoxon rank-sum test, equivalent to the Mann–Whitney U test, with an $\alpha$-value of 1%.

### Evaluation of soft tissue on sensor attachment

We investigate the upper leg, where soft tissue artefacts are considered large^31^. To describe the additional motion effect, we added continuous noise to the synthesised sensor data as suggested by Chèze et al.^52^, with the following motion noise model (N): $\mathsf N = A \, \mathsf \times \, (\mathsf 2 \, \mathsf \pi \, \mathsf f \, \mathsf t \, \mathsf + \, \mathsf \phi )$, where $\mathsf A$ is the noise amplitude, $\mathsf f$ the noise frequency, and $\mathsf \phi$ is the phase angle. The model was chosen to minimise assumptions made about the attachment and soft tissue. We selected reasonable noise model parameters that provide a serious effect on the synthesised sensor data: $A=$ 10% of the maximal value of the simulated sensor’s synthesised acceleration, $\mathsf f=$ 2Hz, as perturbations typically contain the same frequencies as those of the movement^52^, and $\mathsf \phi =180^\circ$.

## Supplementary information

Supplementary information

## Data Availability

Motion data of Case Study 1: “Muscle contributions to mass centre accelerations over a range of running speeds”, Hamner et al., SimTK: https://simtk.org/projects/nmbl_running. Motion data of Case Study 2: “Pre/post FES in post-stroke gait”, Knarr et al., SimTK: https://simtk.org/projects/fesprediction.
