# Supplementary material for: Estimating wearable motion sensor performance from personal biomechanical models and sensor data synthesis
Source: Sci Rep. 2020 Jul 10;10:11450. doi: 10.1038/s41598-020-68225-6 (PMC7351784; doi:10.1038/s41598-020-68225-6)
Supplement: Supplementary file 1 — Supplementary information [file 41598_2020_68225_MOESM1_ESM.pdf]

Supplementary Material

Fig. 10 illustrates the stride duration estimation error distribution per athlete, considering all simulated sensors and all running speeds.

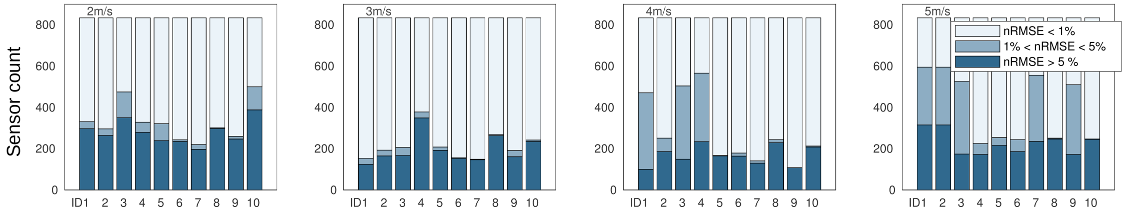

Figure 10. Distribution of nRMSE for all 834 simulated sensors per athlete ID for all running speeds in Case Study 1.

Fig. 11 shows the nRMSE maps for both, *smpl* and *cmplx* algorithms and a selected athlete. From individual athlete maps, it is clearly visible that the *cmplx* algorithm achieves wider map area with low nRMSE, i.e. at the ventral region..

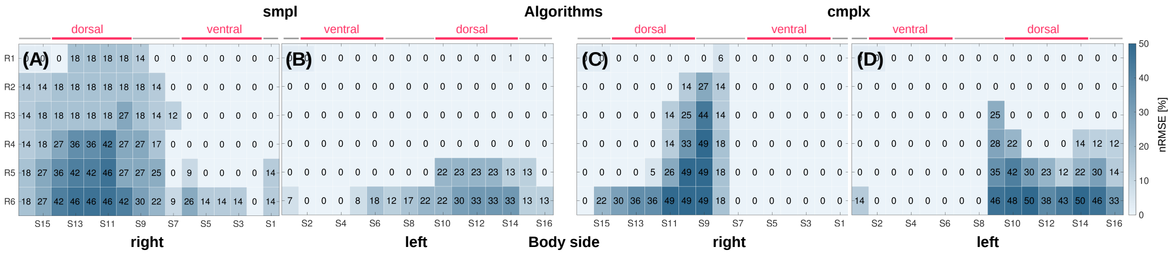

Figure 11. Algorithm comparison for stride duration estimation based on nRMSE maps for running athlete (ID10) running at 5 m/s. **(A,B)**: *smpl* algorithm. **(C,D)**: *cmplx* algorithm.

Fig. 12 shows the sensor count statistics with nRMSE below 10 %. Sensor count differences are significant for the less-affected side between pre- and post-intervention ($p = 0.035$). nRMSE across the study population showed significant differences between affected and less-affected body sides as well as for each side between pre- and post-intervention.

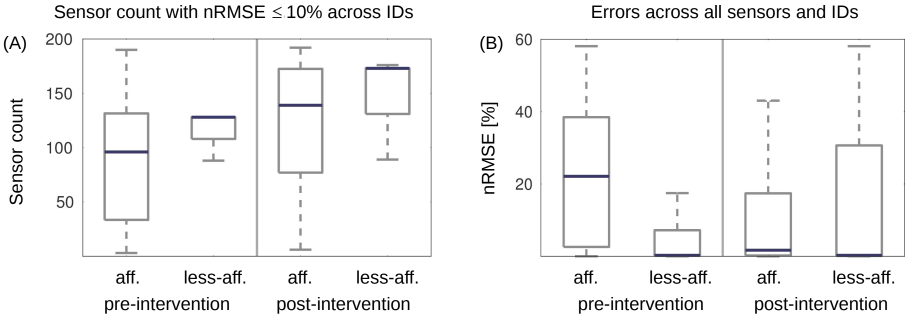

Figure 12. Statistics of the sensor count and errors in Case Study 2, including affected (aff.) and less-affected (less-aff.) body sides during pre- and post-intervention. **(A)**: Sensor count across all patients with nRMSE $\leq$ 10 %. **(B)**: Distribution of nRMSE across all sensors.

An example video of the combined simulation of biomechanical and sensor models for a patient after stroke can be found here: www.cdh.med.fau.de/2019/12/19/estimating-wearable-motion-sensor-performance
